# Monocyte transcriptomes from patients with axial spondyloarthritis reveal dysregulated monocytopoiesis and a distinct inflammatory imprint

**DOI:** 10.1186/s13075-021-02623-7

**Published:** 2021-09-24

**Authors:** Fabian Karow, Biljana Smiljanovic, Joachim R. Grün, Denis Poddubnyy, Fabian Proft, Alice Talpin, Christophe Hue, Anne Boland, Jean-François Deleuze, Henri-Jean Garchon, Ilkay Ergenç, Ann-Sophie De Craemer, Ulrike Erben, Thomas Häupl, Dirk Elewaut, Maxime Breban, Andreas Grützkau, Uta Syrbe

**Affiliations:** 1 Medizinische Klinik für Gastroenterologie, Infektiologie und Rheumatologie, Campus Benjamin Franklin, Charité-Universitätsmedizin Berlin, Hindenburgdamm 30, 12200 Berlin, Germany; 2 Medizinische Klinik für Rheumatologie und Klinische Immunologie, Campus Mitte, Charité-Universitätsmedizin Berlin, Berlin, Germany; 3 Deutsches Rheumaforschungszentrum (DRFZ), A Leibniz Institute, Berlin, Germany; 4 Infection & Inflammation, UMR 1173, Inserm, UVSQ/Université Paris Saclay, Montigny-le-Bretonneux, France; 5 Laboratoire d’Excellence Inflamex, Universite de Paris, Paris, France; 6 CEA, Centre National de Recherche en Génomique Humaine, Université Paris-Saclay, Evry, France; 7 Service de Biochimie, Hôpital Ambroise Paré, Boulogne-Billancourt, France; 8 VIB Center for Inflammation Research, Ghent University Hospital, Ghent, Belgium; 9 Department of Rheumatology, Ghent University, Ghent, Belgium; 10 Service de Rhumatologie, Hôpital Ambroise Paré, Boulogne-Billancourt, France

**Keywords:** Spondyloarthritis, Monocytes, Transcriptomes, Gut translocation

## Abstract

**Background:**

In patients with axial spondyloarthritis (axSpA), monocytes show a pre-activated phenotype. Gut inflammation is a trigger of monocyte activation and may also affect their development in the bone marrow (BM). As gut inflammation is commonly observed in axSpA patients, we performed a detailed analysis of monocyte transcriptomes of axSpA patients in two cohorts and searched for signs of activation and developmental adaptations as putative imprints of gut inflammation.

**Methods:**

Transcriptomes of blood CD14^+^ monocytes of HLA-B27+ axSpA patients and healthy controls (HC) were generated by microarrays from cohort 1 and by RNA-sequencing from cohort 2. Differentially expressed genes from both analyses were subjected to gene set enrichment analysis (GSEA) and to co-expression analysis in reference transcriptomes from BM cells, blood cells and activated monocytes. As serological markers of translocation, 1,3 beta-glycan, intestinal fatty acid binding protein, and lipopolysaccharide binding protein (LBP) were determined by LAL and ELISA.

**Results:**

Transcriptome analysis identified axSpA-specific monocyte signatures showing an imprint of LPS/cytokine-activated monocytes, late granulopoietic BM cells, blood neutrophils, and G-CSF-mobilized blood cells, which suggests LPS/TNF activation and more prominent BM adaptation promoting a neutrophil-like phenotype. GSEA mapped axSpA upregulated genes to inflammatory responses and TNFα signaling and downregulated probe-sets to metabolic pathways. Among translocation markers, LBP levels were significantly increased in axSpA patients vs. HC (*p* < 0.001).

Stratified analysis by disease activity and stage identified an “active disease signature” (BASDAI ≥ 4) with an imprint of LPS/cytokine-activated monocytes and CD16^+^ monocyte subsets. The “AS signature” (vs. non-radiographic axSpA) showed a reinforced neutrophil-like phenotype due to deprivation of dendritic cell transcripts.

**Conclusions:**

The neutrophil-like phenotype of axSpA monocytes points towards a biased monocytopoiesis from granulocyte-monocyte progenitors. This shift in monocytopoiesis and the LPS/cytokine imprint as well as the elevated LBP levels are indicators of systemic inflammation, which may result from bacterial translocation. The BM adaptation is most prominent in AS patients while disease activity appears to be linked to activation and trafficking of monocytes.

**Supplementary Information:**

The online version contains supplementary material available at 10.1186/s13075-021-02623-7.

## Background

Axial spondyloarthritis (axSpA), comprising non-radiographic axial SpA (nr-axSpA) and ankylosing spondylitis (AS), is characterized by inflammation within the sacroiliac joints and the spine.

Although disease development is strongly linked to the presence of the MHC class I molecule HLA-B27 [1], innate immune cells appear particularly important for disease development. Thus, genome wide association studies identified multiple risk genes for AS that are related to innate immune functions such as autophagy, NFkB regulation, and myeloid cell function [2–4]. Moreover, TNFα, which is successfully targeted in axSpA, is predominantly produced by innate immune cells, specifically monocytes [5].

First, Wright et al. reported inflammatory changes in monocytes of AS patients [6]. They found an increase of proinflammatory proteins and an activation of the ubiquitin proteasome pathway in ex vivo isolated AS monocytes by mass spectrometry. In our own previous study in axSpA patients, we observed signs of preactivation and changes in the monocyte subsets [7]. Thus, we found a higher percentage of classical, i.e., CD14^+^CD16^−^ monocytes as compared to healthy controls. The classical monocytes comprise more than 90% of blood monocytes [8] and give rise to the less abundant subsets of intermediate (CD14^+^CD16^+^) and non-classical monocytes (CD14^−^CD16^+^) [9]. As the percentage of individual populations is dynamically changed upon inflammatory triggers such as endotoxemia [9, 10] the change in monocyte subsets in axSpA may be related to monocyte request and activation [9, 10].

In this line, monocytes of axSpA patients showed a pre-activated phenotype as indicated by an increased spontaneous and induced production of proinflammatory cytokines, including TNFα [7].

As monocytes are activated by bacterial stimulation, SpA-comorbidities like inflammatory bowel disease (IBD), subclinical gut inflammation, and psoriasis [11–13] are putative triggers.

A recent study showed that circulating microbial antigens does not only activate monocytes directly but also affect their progenitors in the bone marrow (BM). This results in a shift in progenitor use during monocytopoiesis, in addition to enhanced myeloid cell output [14]. Thus, under homeostatic conditions, classical monocytes comprise a mixture of cells that are derived either from granulocyte-monocyte progenitors (GMP) or monocyte-dendritic cell progenitors (MDP) [15]. GMP-derived monocytes share transcriptional pattern with neutrophils, while MDP-derived monocytes share transcriptional signatures with DCs. It was shown that circulating LPS promotes GMP-dependent generation of neutrophil-like monocytes while CpG favors MDP-dependent monocyte production and by that shifts the ratio between neutrophil-like monocytes and DC-like monocytes [15].

To search for signs of BM adaptation and transcriptional preactivation of monocytes in axSpA patients, we assessed monocyte transcriptomes of axSpA patients. In addition, we determined levels of intestinal fatty acid binding protein (I-FABP), (1→3)-β-D-Glucan (βDG) and lipopolysaccharide binding protein (LBP) as putative serological markers of bacterial translocation.

## Methods

### Patients

Monocyte transcriptomes were generated from two independent cohorts by either GeneChip microarrays (cohort 1) or RNA-sequencing (RNA-seq — cohort 2). Cohort 1 included 25 axSpA patients and 10 healthy controls (HC). Cohort 2 included 32 axSpA patients and 22 HC. All axSpA patients were HLA-B27+ and fulfilled the ASAS criteria of axSpA [16]. AS was defined according to the modified New York criteria [17]. Disease activity was determined by BASDAI [18] and ASDAS [19]. Patients in cohort 1 were treated with non-steroidal anti-inflammatory drugs (NSAIDs) on demand or continuously but not by biological DMARDs; in cohort 2, 34% (11/32 patients) were treated by biological DMARDs. Patient characteristics are given in Table 1.

**Table 1:** Characteristics of axSpA patient cohorts

	Cohort 1			Cohort 2		
	axSpA (*n* = 25)		HC (*n* = 10)	axSpA (*n* = 32)		HC (*n* = 22)
	nr-axSpA (*n* = 10)	AS (*n* = 15)		nr-axSpA (*n* = 9)	AS (*n* = 23)	
Age [years], mean (± SD)	34.4 (± 8.8)	40.4 (± 11.4)	35.5 (± 9.6)	49.2 (± 11.8)	51.4 (± 8.5)	52.5 (± 11.9)
Sex [male], *n* (%)	5 (50)	10 (66.7)	5(50)	5 (56)	15 (65)	2 (9)
Disease duration [years], mean	5.7	10.6	-	26.9	30	-
BASDAI, mean (± SD)	3.8 (± 2.2)	4.2 (± 1.9)	-	4.6 (± 1.6)	3.4 (± 2.3)	-
BASDAI ≥ 4, *n* (%)	3 (30)	9 (60)	-	6 (67)	10 (43)	-
ASDAS-CRP, mean (± SD)	2.0 (± 0.9)	2.3 (± 0.8)	-	n.a.	n.a.	-
CRP [mg/l], mean (± SD)	0.9 (± 0.9)	5.1* (± 6.4)	1.5 (± 1.6)	n.a.	n.a.	-
Uveitis^#^, *n* (%)	1 (10)	3 (20)	-	0 (0)	9 (39)	-
Arthritis^#^, *n* (%)	5 (50)	1 (7)	-	6 (67)	14 (61)	-
Enthesitis^#^, *n* (%)	4 (40)	4 (27)	-	9 (100)	15 (65)	-
Psoriasis^#^, *n* (%)	4 (40)	3 (20)	-	1 (11)	3 (13)	-
IBD^#^, *n* (%)	0 (0)	0 (0)		0 (0)	0 (0)	-

*n.a.* not available

*p < 0.05 vs. nr-axSpA (Mann-Whitney *U* test)

^#^Acute and past manifestations

Analysis of serological translocation markers was performed in cohort 1 and in 59 axSpA patients from a third cohort, the Gent Inflammatory Arthritis and spoNdylitis cohort (GIANT), in which microscopic evaluation of gut biopsies taken upon colonoscopy was performed [20]. None of GIANT patients were treated with biological DMARDs. Gut biopsies were classified according to microscopic evaluation as “no colonic inflammation”, “acute” (neutrophilic) and “chronic” (lymphocytic) inflammation.

All participants gave written consent to the study, which was approved by the local ethical committees of the university hospitals Charité (Berlin, Germany), Ile-de-France XI (Saint-Germaine-en-Laye, France), and Ghent University Hospital (Ghent, Belgium).

### Measurement of translocation markers in the serum

Serum was collected and stored until measurement at least at − 20 to – 80 °C. Commercial ELISA or limulus based detection kits were used for measurement of I-FABP (Hycult Biotech), (1→3)-β-D-Glucan (Fujifilm Wako), and LBP (USCN).

### Monocyte purification, transcriptomic profiling, and statistical analysis

Extended methodological details and details on statistical analysis are given in a supplementary file (Supplementary data 1). In brief, CD14^+^ monocytes were isolated from heparinized whole blood by positive magnetic selection using CD14 microbeads. In cohort 1, RNA isolation and gene chip hybridization were performed as previously described [21]. In cohort 2, monocyte transcriptomes were generated by RNA-sequencing using the Illumina HiSeq4000 sequencer. For GeneChip microarray analysis, significant differences in expression were identified using high performance chip data analysis (HPCDA) (for details see Supplementary data 1). A HPCDA score of > 100 was considered significant. For RNA-sequencing, analysis of differential gene expression was carried out with edgeR using a linear model including the disease status, the gender and the sequencing experiment. Scores reaching a *p*-value < 0.05 were considered significant.

Hierarchical clustering of transcriptomes was performed with Genes@Work software [22]. Principal component analysis (PCA) was performed using Qlucore (Lund, Sweden). For functional allocation, gene set enrichment analysis (GSEA) was performed using the Molecular Signature Database (MSigDB) v7.2 [23, 24]. Differentially expressed probe-sets and genes were analyzed for co-expression in 70 reference transcriptomes (all Affymetrix HG-U133 Plus 2.0 transcriptomes) generated by our own or retrieved from Gene expression omnibus (GEO) data repository as previously described [25]. In brief, data sets were harmonized by quantile normalization before application in co-expression analysis. Pearson correlation coefficients were calculated between the differentially expressed probe-sets of individual comparisons on the basis of the 70 reference transcriptomes. Euclidian distance and average linkage as an agglomeration rule were applied for hierarchical clustering of this gene-to-gene correlation. For analysis of cohort 2, differentially expressed genes were matched to GeneChip probe-sets before analysis.

## Results

### The axSpA-specific monocyte signature shows alterations related to inflammation and metabolism

**Fig. 1:**
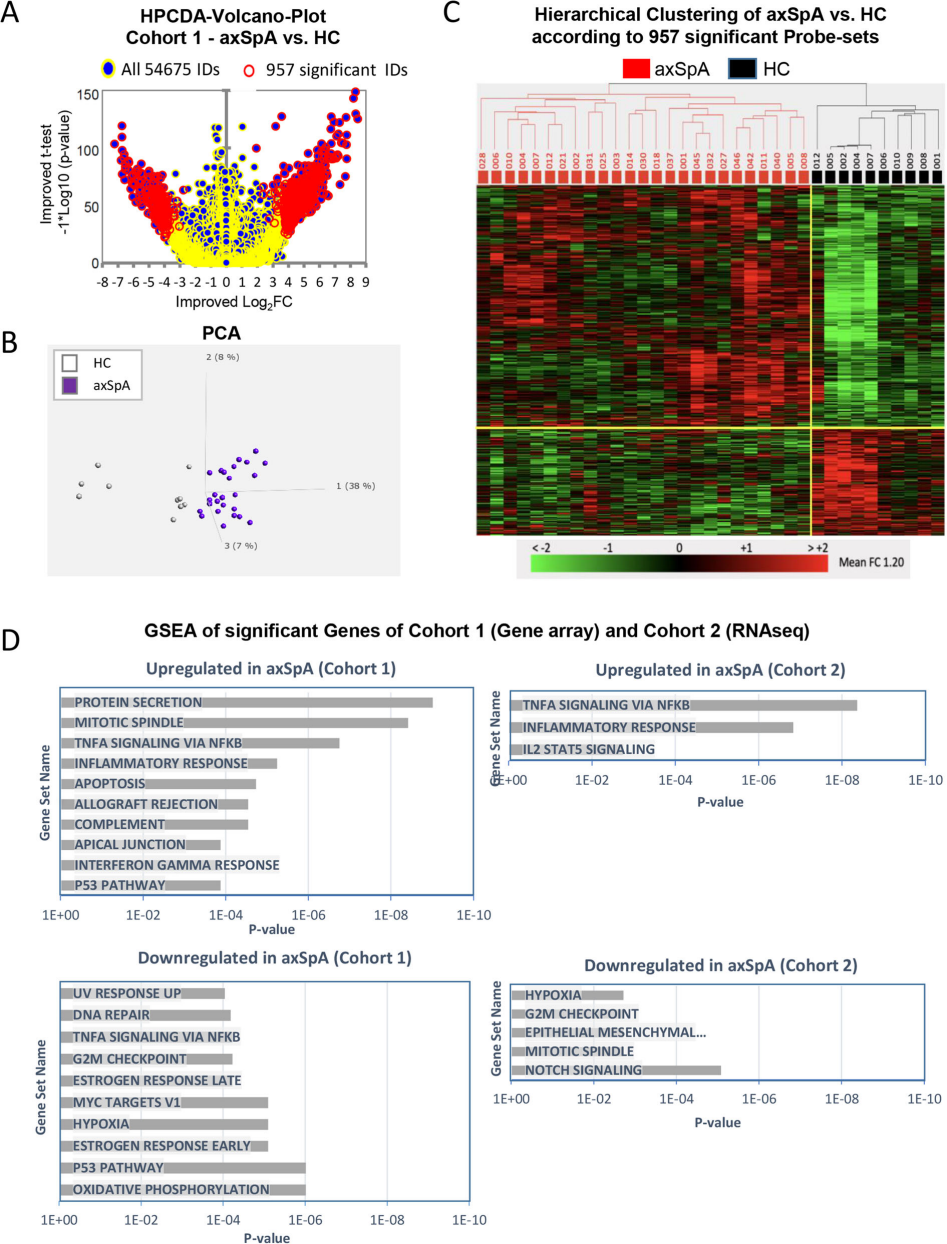
AxSpA signature in monocyte transcriptomes as compared to HC. Monocytes from HLA-B27+ patients with axSpA (*n* = 25) and healthy controls (HC, *n* = 10) of cohort 1 were subjected to GeneChip microarray analysis, which resulted in 957 significant probe-sets according to HPCDA scoring (HPCDA score > 100). **A** 957 significant probe-sets (296 down- and 661 upregulated) shown with HPCDA-volcano plot (red circles). **B** Principal component analysis (PCA) of 25 axSpA patients and 10 HC using the 957 significant probe-sets shows 38%, 8%, and 7% variance in the first 3 principal components 1 (PC1), 2 (PC2), and 3 (PC3). **C** Hierarchical clustering of 25 axSpA patients and 10 HC according to 957 significant probe-sets. Rows represent probe-set, and columns represent individual samples. Row values are color-coded according to their normalized signal intensities (max + 2, min − 2) as indicated by the scale bar (green lower or red color higher than probe-set mean). **D** Gene set enrichment analysis (GSEA) using MSigDB as input data base was performed for the gene lists of upregulated genes (upper panel) and downregulated genes (lower panel) of the axSpA signature of cohort 1 and 2

For transcriptome analysis, blood monocytes were isolated by positive magnetic selection using CD14 microbeads. This selection method resulted in ≥ 95% purity and induces minor transcriptional changes [26]. First, transcriptomes from monocytes of 25 HLA-B27+ axSpA patients and 10 HC from cohort 1 were generated by microarray. Using pair-wise comparisons and a HPCDA score > 100 as cut-off, 957 probe-sets referring to 805 genes were found differentially expressed between axSpA patients and HC (Fig. 1A, Supplementary Table 1). Of these 957 probe-sets, 661 probe-sets were up- and 296 probe-sets were downregulated. Although differences in expression of individual probe-sets were low (mean FC 1.20), both PCA and hierarchical clustering demonstrated clear separation of monocyte transcriptomes of axSpA patients from HC (Fig. 1B, C). For functional classification of differentially expressed genes, the MSigDB as integral part of GSEA was applied. This analysis mapped axSpA upregulated probe-sets with functional groups such as “protein secretion”, “TNFα signaling via NFkB”, “inflammatory responses”, “apoptosis”, and “interferon gamma responses” suggesting a proinflammatory activation of the axSpA monocytes. For axSpA-downregulated genes GSEA showed overlap with pathways such as “oxidative phosphorylation”, “hypoxia”, and “G2M checkpoint” mostly indicative of metabolic changes (Fig. 1D). A few of the downregulated genes also mapped to the “TNF signaling via NFkB” functional group. Most of these genes including *IFNGR2, ETS2,* and *FOS* rather downregulate proinflammatory responses in monocytes/ macrophages [27, 28] suggesting profound alteration of TNF signaling pathway in monocytes in axSpA patients.

In cohort 2, monocyte transcriptomes were generated by RNA-seq. Group-wise comparison of transcriptomes of HLA-B27+ axSpA patients and HLA-B27- HC identified 48 genes as up- and 47 as downregulated (corresponding to 109 and 129 GeneChip probe-sets, respectively,) in axSpA patients compared to HC (*p*-value < 0.05; Supplementary Table 1). The lower number of differentially expressed genes is most likely related to more stringent analysis (HPCDA scoring compared to edgeR computed *p*-values). Furthermore, the overall overlap in significant genes between cohort 1 and 2 was distressingly small (only one gene of the up- and the downregulated gene sets was shared). This is partly explained by limited coverage of RNA-seq genes by the microarrays, which was about 60%. Apart from different methodology, low similarities in individual genes in independent studies analyzing primary tissue are a known problem [23]. However, GSEA can reveal shared pathways between independent studies. Indeed, GSEA of the upregulated genes in cohort 2 overlapped with functional pathways “TNF signaling via NFkB”, “inflammatory responses”, and “IL-2-STAT5 signaling”, which showed strong similarity with cohort 1 functional gene sets. Downregulated genes were enriched in functional groups such as “notch signaling”, “epithelial-mesenchymal transition”, “G2M checkpoint”, and “hypoxia function” according to GSEA which overlapped with cohort 1 in “G2M checkpoint” and “hypoxia” functional groups (Fig. 1D).

Thus, GSEA of both cohorts suggests an imprint of inflammation in axSpA monocytes.

### Co-expression analysis of the axSpA signature genes points towards distinct LPS/TNFα activation and reveals a neutrophil-like phenotype of monocytes

To decipher transcriptional alterations of axSpA monocytes in the context of BM adaptation and LPS/cytokine activation, we applied mapping of monocyte profiles with previously published profiles from GEO data repositories. Up- and downregulated probe-sets are analyzed separately; for analysis of cohort 2 the matched GeneChip probe-sets were used.

The reference profiles comprised (1) 34 transcriptomes of 11 different cell types of early and late myelopoiesis from bone marrow (BM; GSE42519) [29]; (2) 15 transcriptomes from blood cells comprising BDCA1^+^ (*n* = 3) and BDCA3^+^ DCs (*n* = 3), CD15^+^ PMN (*n* = 3) and blood monocyte subsets including classical CD14^+^CD16^−^ monocytes (Mo-CD16^−^; *n* = 3), and non-classical monocytes CD14^−^CD16^+^ (Mo-CD16^+^; *n* = 3) [8] (GSE18565); (3) six transcriptomes of blood leukocytes from healthy donors before and after treatment with G-CSF (GSE7400) [30]; and (4) 12 transcriptomes of blood purified monocytes incubated without stimulus or stimulated with TNFα, LPS, IFNγ, or IFNα (GSE38351) [21, 31].

By applying the differentially expressed probe-sets to these transcriptomes, their regulation during myelopoiesis, in subsets of blood cells and upon in vitro activation of monocytes can be studied. The list of differentially expressed probe-sets is retrieved from the reference transcriptomes; quantile normalized and co-regulated probe-sets are identified by calculating Pearson correlation coefficients. Hierarchical clustering of this gene-to-gene correlation (correlation matrix) provides the order for display of the reference transcriptomes showing relative expression values. This allows visual identification of co-expression clusters among the differentially expressed probe-sets in the reference transcriptomes.

**Fig. 2:**
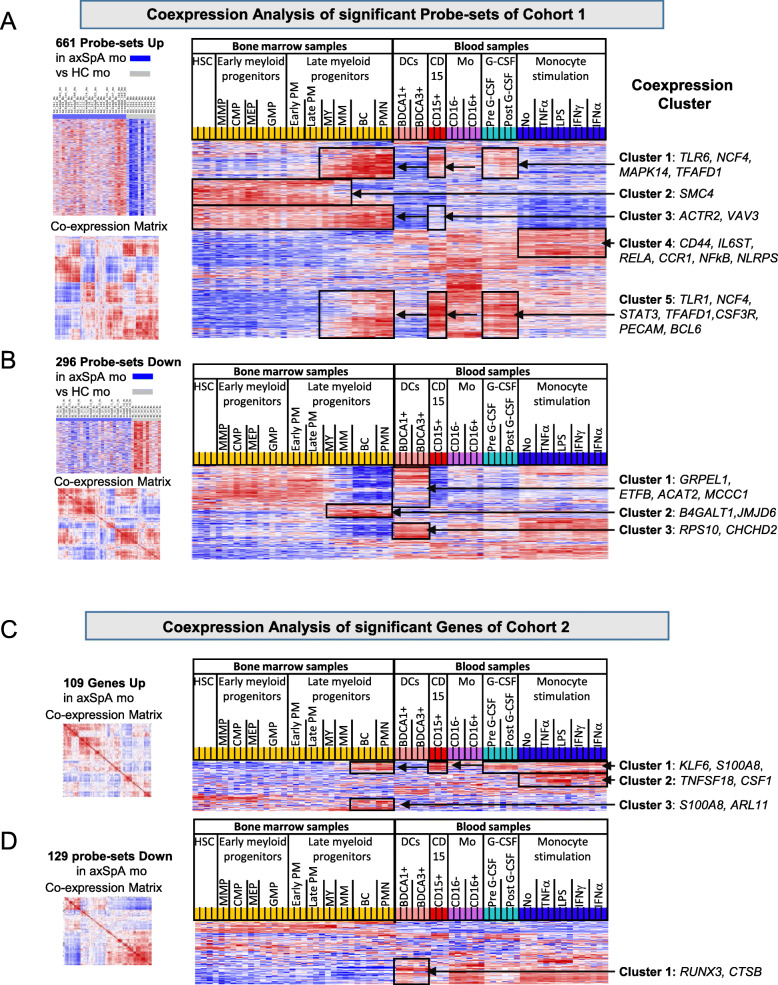
Co-expression profiling of axSpA signature probe-sets in reference transcriptomes. Co-expression profiling of up- and downregulated axSpA signature probe-sets of microarray analysis of cohort 1 (**A** + **B**) and axSpA signature genes of RNA-seq analysis of cohort 2 matched to microarray probe-sets (**C** + **D**) in reference transcriptomes. Reference transcriptomes comprised transcriptomes from (1) bone marrow (including hematopoietic stem cells (HSC), early and late myeloid progenitors, and polymorph nuclear neutrophils (PMN)), (2) blood cells (comprising BDCA1^+^ and BDCA3^+^ dendritic cells (DCs), CD15^+^ neutrophils, CD16^−^, and CD16^+^ monocytes), (3) blood leukocytes before and after G-CSF treatment, and (4) monocytes stimulated in vitro with or without the indicated cytokines. Up- or downregulated signature probe-sets lists were applied to the reference transcriptomes. After quantile normalization, the relative signal intensities from the reference transcriptomes were correlated and obtained correlation coefficients were hierarchically clustered as shown by the co-expression matrixes. This probe-set order was applied to display relative probe-set intensities of the 70 reference transcriptomes. Red indicated increased signal expression (max = 2) or positive correlation (max = 1) and blue indicates decreased signal expression (min = − 2) or negative correlation (min = − 1). Clusters of co-expression are boxed and examples of genes within individual clusters are given on the right

Among the probe-sets upregulated in axSpA patients of cohort 1, we found five clusters with distinct co-expression in BM and blood cells (C1-C5, Fig. 2A). Most strikingly, genes of cluster 1–3 and cluster 5 were overrepresented in late stage granulocytopoiesis and PMNs but weakly expressed in DCs. Moreover, cluster 1 and 5 probe-sets were also enriched after G-CSF treatment. These co-expression cluster included genes such as *TLR6*, *CCR1*, *NFkB*, *STAT3,* and *CSF3R* which are related to inflammatory and PMN effector functions such as bacterial recognition, ROS production and phagocytosis [32].

In addition, a co-expression cluster comprising fewer genes was found in in vitro activated monocytes (cluster 4). GSEA of genes in this cluster indicated overlap with TNF signaling via NFkB, IL-6-JAK-STAT3-signaling and inflammatory responses among others.

Co-expression analysis of the 296 downregulated probe-sets of the axSpA signature of cohort 1 revealed three clusters (Fig. 2B). Most strikingly, genes of cluster 1 and 3, comprising > 50% of the downregulated probe-sets, were highly expressed in DCs but strongly downregulated in PMNs and late granulocytopoiesis. GSEA of these cluster genes revealed overlap with metabolic pathways such as oxidative phosphorylation, cholesterol homeostasis, and adipogenesis, cell cycle control, and mTORC1 signaling. These metabolic pathways are constitutively active in resting DCs while PMNs relay on glycolytic pathways. Genes of cluster 2, comprising less than 10% of the downregulated genes in axSpA, were upregulated in late granulopoiesis and related to TNFα signaling and hypoxia.

Co-expression profiling of significant genes of cohort 2 revealed similar changes as in cohort 1. Thus, co-expression of the upregulated genes was found in late granulocytopoiesis and blood PMNs as well as in LPS/cytokine stimulated monocyte signatures. Among the downregulated genes a co-expression cluster was found in DCs (Fig. 2C, D). Genes of this cluster were in opposite downregulated in PMNs and late granulocytopoiesis.

Altogether, upregulated genes of the axSpA signature are found in late PMNs of the BM, in blood PMNs, and in response to G-CSF while downregulated genes are DC associated. Thus, axSpA monocytes exhibit a neutrophil-like phenotype but deprivation of DC features which is compatible with a shift towards GMP-dependent generation of monocytes. Together with the LPS/TNF imprint, this may reflect response and adaptation to continuous microbial stimulation possibly originating from the gut.

### LBP serum levels are increased in axSpA patients, predominantly in those with chronic gut inflammation

To assess putative microbial translocation we measured serum concentration of I-FABP, βDG and LBP in patients of cohort 1, in which none of the patient suffered from clinical IBD. Pathological elevations of these parameters were reported before in clinical conditions of intestinal inflammation such as coeliac disease, HIV enteropathy, and Crohns disease [33–35].

**Fig. 3:**
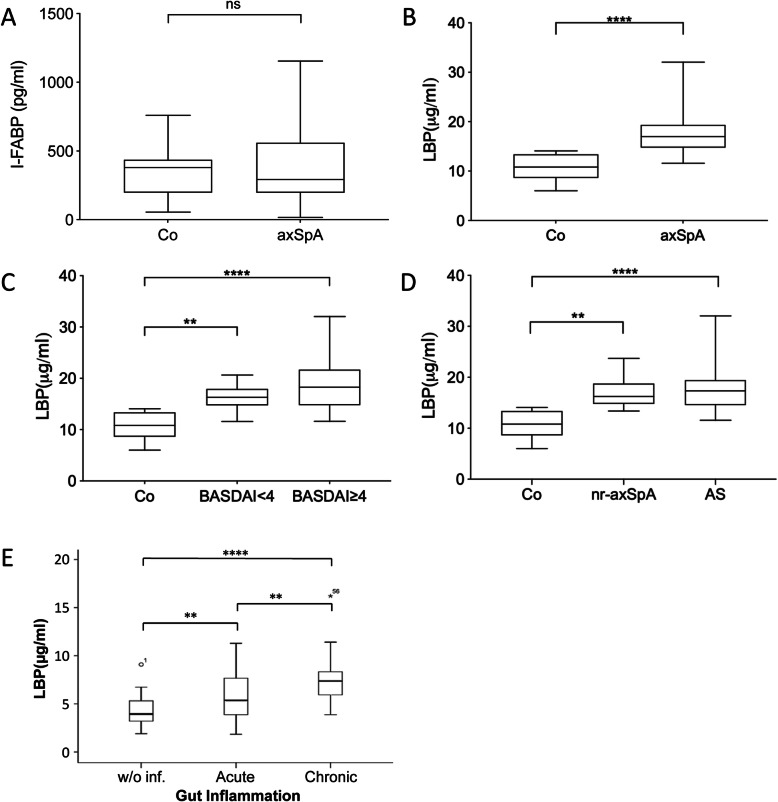
Translocation markers in serum of axSpA patients. I-FABP and LBP levels were determined by ELISA in serum samples of axSpA patients and healthy controls (HC). **A** I-FABP levels in axSpA patients (*n* = 29) and HC (*n* = 11) of cohort 1. **B** LBP serum levels in axSpA patients (*n* = 29) compared to HC (*n* = 11) of cohort 1. **C** LBP levels in axSpA patients stratified according to disease activity into patients with active disease defined by BASDAI ≥ 4 (*n* = 19) and patients with inactive disease (BASDAI < 4, *n* = 13). **D** LBP levels in patients classified as non-radiographic axSpA (nr-axSpA, *n* = 10) and patients classified as ankylosing spondylitis (AS, *n* = 19) of cohort 1. **E** LBP levels in axSpA patients of cohort 3 (GIANT cohort) grouped according to microscopic gut inflammation graded as no inflammation (w/o. inf.; *n* = 30), acute (*n* = 25), and chronic inflammation (*n* = 25). In **A**–**D**, Mann-Whitney *U* test was used for two group comparisons and Kruskal-Wallis test and Dunn’s post test for multiple group testing. In **E**, *T* test was used (after confirmed normal distribution). *p < 0.05, **p < 0.01, ***p < 0.001, ****p < 0.0001

As a result, we found no difference in I-FABP serum levels between axSpA and HC (Fig. 3A) and βDG levels were undetectable in both axSpA and HC samples (while pathological elevation was found in patients with metabolic syndrome in our hands; unpublished observations B.S.). However, LBP serum levels were significantly higher in axSpA patients compared to HC (*p* < 0.001; Fig. 3B). LBP levels tended to be higher in patients with active disease (BASDAI > 4) although this did not reach statistical difference (Fig. 3C) and did not differ between nr-axSpA and AS patients (Fig. 3D).

To determine whether LBP levels are related to intestinal inflammation in axSpA patients, we analyzed LBP levels in axSpA patients of the GIANT cohort in whom presence of intestinal inflammation was assessed by colonoscopy and microscopic evaluation. Interestingly, LBP levels were higher in patients with microscopic gut inflammation than in patients without gut inflammation. The highest LBP levels were found in patients with chronic gut inflammation suggesting a relation between LBP levels and gut inflammation in axSpA patients (Fig. 3E).

### “Active-axSpA” monocyte signature reveals association with inflammatory response, gene pattern of terminally differentiated CD16^+^ blood monocytes, and suppression of DC-associated genes

**Fig. 4:**
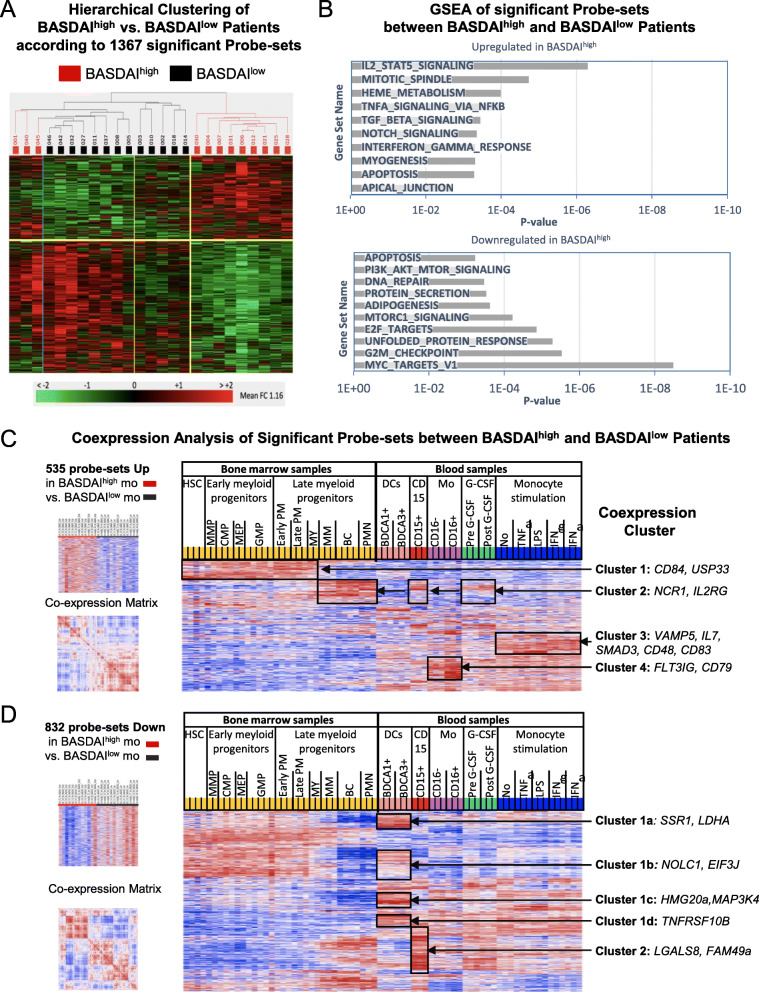
“Active disease” signature in monocyte transcriptomes of axSpA patients. Monocyte transcriptomes of patients (of cohort 1) with active axSpA according to a BASDAI ≥ 4 (BASDAI^high^) were compared to those of patients with inactive disease (BASDAI < 4; BASDAI^low^). **A** Hierarchical clustering of active and inactive axSpA patients according to 1367 significantly different probe-sets (HPCDA score > 100). **B** Gene set enrichment analysis (GSEA) using MSigDB as input database identified overlap of upregulated and downregulated genes with the indicated pathways. **C** Co-expression analysis in reference transcriptomes performed as in Fig. 2 of upregulated probe-sets of the comparison between BASDAI^high^ and BASDAI^low^ patients. **D** Co-expression analysis in reference transcriptomes of downregulated probe-sets in BASDAI^high^ patients. Red indicated increased signal expression (max = 2) or positive correlation (max = 1) and blue indicates decreased signal expression (min = − 2) or negative correlation (min = − 1). Clusters of co-expression are boxed and examples of genes within individual clusters are given on the right

To determine, if disease activity is associated with distinct transcriptional changes in monocytes, we compared transcriptomes of axSpA patients from cohort 1 separated into active patients (BASDAI ≥ 4 = BASDAI^high^, *n* = 12) and inactive patients (BASDAI < 4 = BASDAI^low^, *n* = 13). The group-wise comparison of these two groups of patients identified 1367 probe-sets differentially expressed (HPCDA score > 100). Hierarchical clustering showed the heterogeneity between patients but very distinctive separation of nine BASDAI^high^ patients from eight BASDAI^low^ patients, while three BASDAI^high^ patients clustered outside these groups and five BASDAI^low^ patients clustered in-between these groups (Fig. 4A).

Out of 1367 probe-sets of this “active axSpA signature”, 535 were up- and 832 were downregulated in monocytes of BASDAI^high^ patients. GSEA of upregulated genes identified associations with inflammatory pathways such as “IL-2-STAT5”, “TNFα”, “TGFβ”, and “Notch signaling”, as well as with “IFNγ response” among other pathways. The downregulated genes showed an overlap with functional groups such as “myc target”, “unfolded protein response”, and “adipogenesis” (Fig. 4B).

Up- and downregulated genes of the “active axSpA signature” were analyzed for co-expression in 70 reference transcriptomes as described above. For the upregulated genes, in total four gene clusters were identified, where cluster 1 and 2 genes showed co-expression in early and late granulocytopoiesis, cluster 3 in LPS/cytokine-activated monocytes and cluster 4 showed distinct co-expression in the non-classical CD16^+^ monocyte subset in the blood.

GSEA of cluster 3 und 4 genes indicated overlap with functional pathways “interferon gamma response”, “IL2-STAT5 signaling”, “inflammation response”, “TNFα signaling via NFkB”, “TGFβ signaling”, “mTORC1”, and “Notch signaling”.

In contrast, among the 832 probe-sets downregulated in BASDAI^high^ patients (and hence upregulated in BASDAI^low^ patients), high expression was found in DCs (cluster 1a–d; Fig. 4D). Genes of this cluster were underrepresented in PMNs and progressively downregulated in late granulocytopoieses. GSEA revealed overlap with metabolic pathways such as “mTORC1” (cluster 1a), “Myc targets”, “G2M checkpoint”, “E2F targets”, “DNA repair”, and “adipogenesis” (cluster 1b). A small co-expression cluster was found in PMNs (cluster 2).

Thus, the “active SpA signature” points towards LPS/cytokine activation, changes in monocyte subset mobilization (CD16^+^-associated gene enhancement of non-classical monocytes) and suppression of DC-associated genes in monocytes of active axSpA patients.

### Monocyte transcriptomes of nr-axSpA and AS patients differ in pathways controlling metabolism in DCs

Nr-axSpA and AS are considered consecutive stages of axSpA differing in presence of structural damage. However, progression to the radiographic stage is slow and limited to a small group of patients [36] suggesting that genetic heterogeneity among patients may determine progression to AS rather than time.

**Fig. 5:**
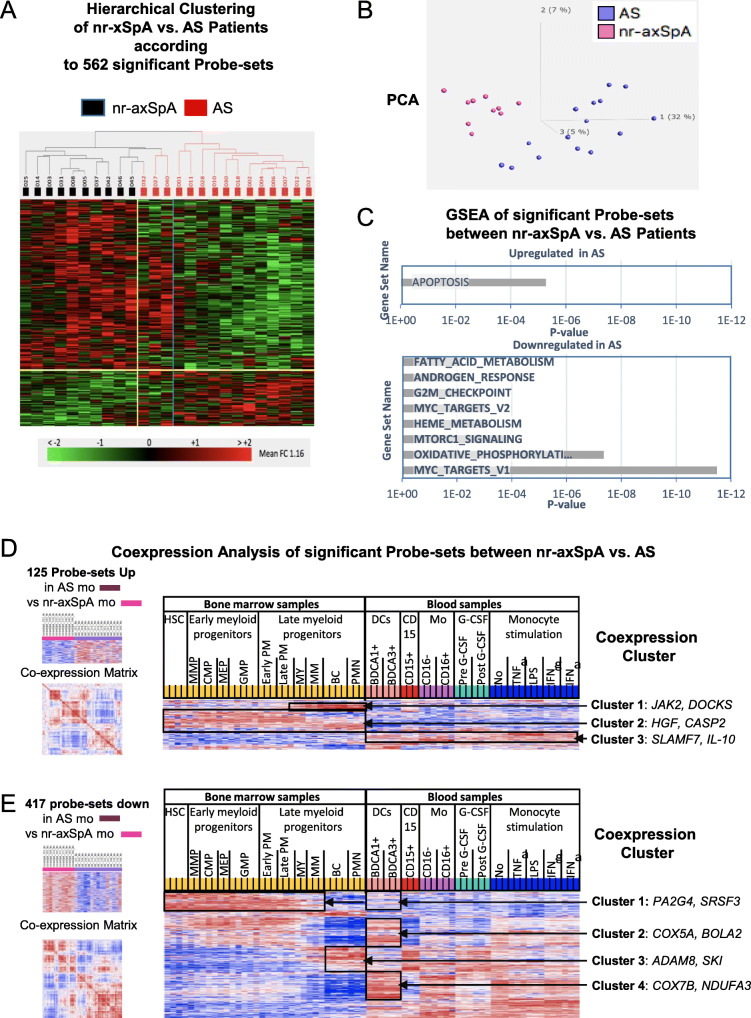
Differences in monocyte transcriptomes between nr-axSpA and AS patients. Five hundred and sixty-two significant probe-sets (HPCDA score > 100) were identified by comparing monocyte transcriptomes of nr-axSpA patients (*n* = 10) and AS patients (*n* = 15) of cohort 1. **A** Hierarchical clustering and **B** principal component analysis using 562 significant differentially expressed probe-sets of 10 nr-axSpA patients and 15 AS patients. **C** Overlap of upregulated and downregulated probe-sets with the indicated functional groups according to GSEA based on MSigDB as input database. **D** Co-expression analysis performed as in Fig. 2 of probe-sets upregulated in AS patients in reference transcriptomes from bone marrow and blood cells. **E** Co-expression analysis of probe-sets downregulated in AS patients in reference transcriptomes. Red indicated increased signal expression (max = 2) or positive correlation (max = 1) and blue indicates decreased signal expression (min = − 2) or negative correlation (min = − 1)

Since monocytes give rise to osteoclast which promote bone destruction in AS [37], we performed pair-wise comparisons of transcriptomes of nr-axSpA and AS patients of cohort 1 to search for an AS-specific monocyte transcriptomic profile. In total, 562 probe-sets were identified to be differentially expressed, again with low magnitudes of changes in their expression. Hierarchical clustering demonstrated that all 10 nr-axSpA clustered separately from the AS group, but 3 AS clustered with the nr-axSpA patients (Fig. 5A). PCA with these 562 significant probe-sets confirmed distinctive differences in monocyte transcriptomes of 10 nr-axSpA and 15 AS patients (Fig. 5B).

GSEA of probe-sets upregulated in AS showed overlap with the functional group “apoptosis”, while probe-sets downregulated in AS patients mapped to metabolic pathways such as oxidative phosphorylation, myc and mTORC1 (Fig. 5C).

Co-expression analysis of the 145 upregulated AS signature probe-sets revealed co-expression of cluster 1 and 2 in early and late myelopoiesis (Fig. 5D). In contrast, probe-sets downregulated in AS (Fig. 5E) where clearly overrepresented in DCs (cluster 1, 2, and 4) and repressed in PMNs and late granulocytopoiesis. Only genes of cluster 3, comprising less than 25% of the downregulated probe-sets, were higher expressed in late granulocytopoiesis and downregulated in DCs (cluster 3). Cluster 1, 2, and 4 genes overlapped with metabolic pathways such as “myc pathway”, “unfolded protein response”, and “oxidative phosphorylation”.

Thus, monocytes of AS patients show a stronger shift towards a neutrophil-like phenotype as compared to nr-axSpA patients which express more DC-like features suggesting a stronger adaptation of monocytopoiesis in the BM of AS patients.

## Discussion

Gut inflammation is a common comorbidity in axSpA; however, the impact on systemic immunity is poorly understood. Monocytes as sensors of microbes might be particularly affected and previous studies reported a proinflammatory, i.e., activated phenotype of monocytes in axSpA patients [6, 7]. To study this monocytic activation in more detail, we examined transcriptional pattern from monocytes of axSpA patients for signs of activation and developmental adaptations.

As a result of pathway and co-expression analysis of differentially expressed genes from two independent cohorts, we found an “axSpA monocyte signature” showing a small, but distinct, LPS/TNF imprint and more prominent signs of BM adaptation. Specifically, we found an enrichment of inflammatory pathways such as “TNF signaling”, “inflammatory response”, “allograft rejection”, and “interferon gamma” in the axSpA signature. However, co-expression analysis using reference transcriptomes of myelopoietic cells, blood cell subsets, and in vitro activated monocytes placed these transcriptional changes into the context of a shifted monocytopoiesis. In fact, only a small part of the axSpA signature genes was found to be regulated by LPS/cytokine treatment in monocytes. In contrast, there was co-expression of upregulated axSpA signature genes in late granulocytopoietic BM cells, BM PMNs, and CD15^+^ blood PMNs while downregulated axSpA genes were DC-associated genes. Thus axSpA monocytes have a neutrophil-like phenotype with deprivation of DC features compatible with a skewing of monocytopoiesis towards GMP-driven monocytopoiesis [15]. Similarly, an expansion of GMP-driven hematopoiesis was recently described in experimental SpA in SKG mice [38]. The severe inflammation of the small intestine that accompanies SpA induction in this model is considered the stimulus for the changes in hematopoiesis.

In this study here, none of the axSpA patients suffered from overt IBD and of translocation markers we only found increased LBP levels, but no increase of I-FABP and βDG levels. However, in axSpA cohort 3 with histological assessment of gut inflammation, we found highest LBP levels in patients with signs of chronic gut inflammation suggesting a relation of LBP levels to gut pathology in axSpA. As LBP levels are controlled even under homeostatic conditions by gut microbiota [39], they appear more sensitive to changes in gut permeability than for instance I-FABP. LBP is also an acute phase protein like CRP [40]; however, in axSpA patients, we found elevated LBP levels without elevation of CRP in most patients. Both, the elevated LBP levels but also the transcriptional axSpA signature of monocytes with signs of BM adaptation and LPS/TNF activation are compatible with low level but chronic bacterial translocation in these patients. Alternatively, a direct effect of HLA-B27 on monocyte development and hematopoiesis could be discussed and needs experimental verification.

A methodological issue in our study is the low overlap of individual genes in transcriptomic analysis of cohort 1 and 2. This may be related to differences in molecular techniques (geneChip vs. RNA-seq) [41], data analysis (HPCDA scoring vs. edgeR based scoring), and cohort population (age, male/female matching, use of biologics). A low overlap of individual genes in transcriptomes from primary tissue from independent studies, even if standardized by technology, is a known hurdle and pathway analysis using GSEA or co-expression analysis can be used to detect functional similarities between signatures [23]. In this line, GSEA found great overlap in functional groups in particular of upregulated genes between both cohorts and co-expression analysis revealed the abovementioned LPS/TNF imprint and signs of BM adaptation in both cohorts. However, even though GSEA and co-expression analysis link both studies further confirmation of the transcriptional axSpA signature for instance by protein expression or single cell profiling of monocytes is required and would significantly enhance the relevance of our findings. Furthermore, additional studies should compare monocyte transcriptomes between different rheumatic and inflammatory disease to better define the specificity of the signature.

To determine if disease activity in axSpA is associated with transcriptional changes in monocytes we compared transcriptomes from active (BASDAI ≥ 4) vs. inactive patients (BASDAI < 4) in cohort 1, in which none of the patients was treated with biologics. The “active axSpA signature” resulted in only reasonable clustering of active and inactive patients, which is not unexpected given that BASDAI is patient-reported without molecular parameters of inflammation. However, “active axSpA signature” genes were enriched in inflammatory pathways such as IL-2-STAT5 signaling, TNFα signaling, and interferon gamma response while downregulated pathways comprised metabolic pathways. Distinct co-expression of the upregulated “active axSpA” genes was found in LPS/cytokine-activated monocytes, G-CSF mobilized cells and in CD16^+^ non-classical monocytes. This CD16^+^ non-classical monocyte pattern may even be underrepresented in our study since we enriched for CD14 which is lower expressed on CD16^+^ cells. Non-classical CD16^+^ monocytes usually recirculate in blood while classical CD16^−^ monocytes migrate into peripheral tissues [9]. In view of the co-signature with G-CSF mobilized cells, the enrichment of the CD16^+^ non-classical monocyte pattern may reflect enhanced monocyte mobilization and enhanced extravasation of classical monocytes, for instance into the gut, in active axSpA.

We also compared transcriptomes of patients classified as nr-axSpA and AS which are considered successive stages of axSp A[42]. Interestingly, we found an “AS monocyte signature” which clearly separated most AS from nr-axSpA patients. Upregulated genes were coexpressed in myelopoiesis and involved apoptosis pathways while downregulated genes were highly expressed in DCs but strongly suppressed in PMNs and late granulocytopoietic cells. These genes are enriched in metabolic pathways such as fatty acid metabolism, mTORC1 signaling and oxidative phosphorylation used by resting DCs but not PMNs for energy consumption [43, 44]. This deprivation of resting DC features in AS patients suggests a stronger BM adaptation in AS patients towards GMP-driven monopoiesis. Interestingly and in line with similar clinical disease activity in AS and nr-axSpA patients, no signs of enhanced acute inflammatory responses (i.e., monocyte mobilization and LPS/TNF response) are found in the AS signature.

Changes in monocytopoiesis may also affect development of structural damage as osteoclasts derived from monocytes. In AS patients, overactivity of RANK-mediated osteoclastogenesis was found related to sacroiliac joint ankylosis [45]. Favored oxidative phosphorylation in nr-axSpA monocytes may result in lower bone destruction as oxidative phosphorylation is linked to reduced osteoclastic degradation and glycolysis to osteoclastic destruction [46].

## Conclusions

In conclusion, we here describe changes in monocyte transcriptomes of axSpA patients which are compatible with LPS/TNF activation of monocytes and a more prominent adaptation of monocytopoiesis resulting in a shift from MDP towards GMP-driven monopoiesis. Together with the LBP increase in axSpA patients, this may result from low-grade translocation from the gut. These findings provide a mechanistic link between gut inflammation and bone marrow, the site of skeletal inflammation and destruction.

## Supplementary Information

**Additional file 1.** Material and Methods

**Additional file 2.** List of differentially expressed probe-sets (HPCDA-Score > 100) of Cohort 1 (GeneChip). List of differentially expressed probe-sets (p<0.5) of Cohort 2 (RNASeq)

## Data Availability

The datasets used and/or analyzed during the current study are available from the corresponding author on reasonable request.

## Abbreviations

axSpA: Axial spondyloarthritis
BM: Bone marrow
HC: Healthy controls
LBP: Lipopolysaccharide binding protein
nr-axSpA: Non-radiographic axial SpA
AS: Ankylosing spondylitis
IBD: Inflammatory bowel disease
GMP: Granulocyte-monocyte progenitors
MDP: Monocyte-dendritic cell progenitors
I-FABP: Intestinal fatty acid binding protein
LBP: lipopolysaccharide binding protein
βDG: (1→3)-β-D-Glucan
RNA-seq: RNA-sequencing
NSAIDs: Non-steroidal anti-inflammatory drugs
GIANT: Gent Inflammatory Arthritis and spoNdylitis cohort
HPCDA: High performance chip data analysis
PCA: Principal component analysis
GSEA: Gene set enrichment analysis
MSigDB: Molecular Signature Database
GEO: Gene expression omnibus
