# Supplementary material for: Monocyte transcriptomes from patients with axial spondyloarthritis reveal dysregulated monocytopoiesis and a distinct inflammatory imprint
Source: Arthritis Res Ther. 2021 Sep 24;23:246. doi: 10.1186/s13075-021-02623-7 (PMC8461951; doi:10.1186/s13075-021-02623-7)
Supplement: Supplementary file 1 — Additional file 1. Material and Methods [file 13075_2021_2623_MOESM1_ESM.pdf]

Supplementary File 1:

Material and Methods:

Monocyte purification and transcriptomic profiling

Monocytes from heparinized whole blood were isolated by positive magnetic selection using CD14 MicroBeads (MACS WholeBlood Column Kit, Miltenyi Biotec). The purity of $CD14^+$ cells was at least 95%. Cells were lysed in RLT buffer (Qiagen) and stored at -80°C before RNA extraction.

In cohort 1, mRNA profiling was performed by GeneChip microarrays (Affymetrix HG-U133 Plus 2.0, Thermo Fisher Scientific). RNA isolation, gene-chip hybridization and quality checks were performed as previously described (Smiljanovic et al., 2010).

In cohort 2, monocyte transcriptomes were generated by RNA-sequencing. Sequencing libraries were prepared from high quality RNA (RIN>8) using the Illumina TruSeq® stranded total RNA protocol. Libraries were then sequenced paired-ended using an Illumina HiSeq4000 sequencer to generate an average of 65 million of uniquely mapped and properly paired reads per sample.

Statistical analysis

For GeneChip microarray analysis, CEL-files were normalized with Robust Multichip Averaging (RMA) algorithm using RMAExpress with background correction, quantile normalization, and Median Polish as summarization. This quantile normalization was done for each comparison separately. To determine significant differences in expression between two groups high performance chip data analysis (HPCDA) was performed according to Menssen et al. (Menssen et al., 2009). In group-wise comparisons each transcriptome of one group was compared to each transcriptome from the other group (i. e. 250 comparisons for 25 axSpA vs. 10 HC transcriptomes). Genes with a HPCDA score of > 100 and all values described in Menssen et al. (Menssen et al., 2009) were considered significant. This empirically developed HPCDA

score includes all values calculated with HPCDA, as Fold Change, signal differences between two groups, four different t tests, presence or absence of each gene, Increased or Decreased calls for every chip to chip comparison, high and low confidence intervals and HPCDA-Volcano plot x and y values (compare Table 1 at the end and further explanations in DOI: 10.13140/2.1.4942.9289 or DOI: 10.13140/RG.2.1.1426.8321). This analysis promotes detection of small changes between samples which helps in identifying context-specific pattern.

For RNA-sequencing, RNA-seq reads were aligned with the splice-aware STAR aligner on the human genome (GRCh38) and transcriptome (gencode v28) after adapter and low-quality bases trimming. Uniquely mapping fragments were assigned to exons and genes with featureCounts. Counts data were normalized using the TMM (trimmed means of M-values) method. Analysis of differential gene expression was carried out with edgeR, using a linear model including the disease status, the gender and the sequencing experiment. Scores reaching a p-value < 0.05 were considered significant.

Hierarchical clustering of transcriptomes using differentially expressed probe-sets was performed with Genes@Work software which applies Pearson correlation with z normalized gene vectors as similarity/distance measure and center of mass as cluster type (Lepre et al., 2004). Principal component analysis (PCA) was performed using Qlucore (Lund, Sweden).

Functional allocation and co-expression analysis of differentially expressed genes

For functional allocation, gene set enrichment analysis (GSEA) using the pre-ranked list of differentially expressed probe-sets (GeneChip) or genes (RNA-seq) was performed using the GSEA software (Broad Institute) and the annotated gene sets of

the Molecular Signature Database (MSigDB) v7.2 (Subramanian et al., 2005; Mootha et al., 2003).

In addition, differentially expressed probe-sets identified in comparisons between 1) axSpA versus HC, 2) $BASDAI^{high}$ vs. $BASDAI^{low}$ and 3) nr-axSpA versus AS were analyzed for co-expression in 70 reference transcriptomes (all Affymetrix HG-U133 Plus 2.0 transcriptomes) generated by our own or retrieved from Gene expression omnibus (GEO) data repository.

The reference transcriptomes included (1) 34 transcriptomes of 11 different cell types of early and late myelopoiesis from bone marrow (BM; GSE42519) (Rapin et al., 2014), (2) 15 transcriptomes from blood cells comprising $BDCA1^+$ (n=3) and $BDCA3^+$ DCs (n=3), $CD15^+$ PMN (n= 3) and blood monocyte subsets including classical $CD14^+CD16^-$ monocytes (Mo-$CD16^-$; n=3), and non-classical monocytes $CD14^-CD16^+$ (Mo-$CD16^+$; n=3) (Ziegler-Heitbrock et al., 2010) (GSE18565), (3) six transcriptomes of blood leukocytes from healthy donors before and after treatment with G-CSF (GSE7400) (Buzzeo et al., 2007) and (4) 12 transcriptomes of monocytes isolated from peripheral blood and incubated for 90 minutes without stimulus or stimulated with TNFα, LPS, IFNγ or IFNα (GSE38351) (Smiljanovic et al., 2012).

For the coexpression analysis, 70 reference transcriptomes were utilized and Pearson correlation coefficients were calculated between signals of all differentially expressed probe-sets. Hierarchical clustering of this gene-to-gene correlation matrix was performed by applying Euclidean distance and average linkage as an agglomeration rule as previously described (Smiljanovic et al., 2020). The order of probe-sets determined by these calculations was used to determine co-expressed probe-sets among the 70 reference transcriptomes. To harmonize data from different studies, all transcriptomes were quantile normalized and subsequently applied for co-expression analysis.

Intensity of expression is color-coded – red indicated increased signal expression (max = 2) or positive correlation (max=1) and blue indicates decreased signal expression. Cluster of coexpression were visually depicted and boxed in graphical depictions.

Symbols, cited in paper			axSpA_vs_HC (49 of 957 significant IDs)							AS_vs_nr-axSpA (13 of 562 significant IDs)							BASDAI_high_vs_BASDAI_low (42 of 1367 significant IDs)							AS_vs_HC (50 of 1584 significant IDs)						
Symbol	Affy_ID	Gene_Name	Signal Differ	FC	% Incr - Decr	ttest SLREvB vs. BvBEvE	HPCDA Score	HPCDA VolcanoX	HPCDA VolcanoY	Signal Differ	FC	% Incr - Decr	ttest SLREvB vs. BvBEvE	HPCDA Score	HPCDA VolcanoX	HPCDA VolcanoY	Signal Differ	FC	% Incr - Decr	ttest SLREvB vs. BvBEvE	HPCDA Score	HPCDA VolcanoX	HPCDA VolcanoY	Signal Differ	FC	% Incr - Decr	ttest SLREvB vs. BvBEvE	HPCDA Score	HPCDA VolcanoX	HPCDA VolcanoY
TLR6	207446_at	toll-like receptor 6	15.62	1.13	54.00	3.201E-27	543.805	5.437	62.087															17.90	1.15	61.33	5.610E-27	1931.398	6.711	61.695
MAPK14	202530_at	mitogen-activated protein kinase 14	114.80	-1.10	50.40	3.353E-27	377.527	-5.035	63.466																					
MAPK14	210449_x_at	mitogen-activated protein kinase 14	76.08	1.13	48.80	7.546E-23	150.760	4.259	53.247															81.75	1.13	50.67	4.568E-17	236.464	4.672	40.039
MAPK14	211087_x_at	mitogen-activated protein kinase 14																						26.16	1.12	48.00	1.890E-13	221.588	4.556	30.676
MAPK14	211561_x_at	mitogen-activated protein kinase 14	59.49	1.11	46.40	4.499E-24	198.937	4.430	55.714															68.12	1.12	50.67	2.367E-19	527.809	5.329	45.035
ACTR2	1554390_s_at	ARP2 actin-related protein 2 homolog (yeast)	236.77	1.17	56.00	5.343E-26	524.112	5.481	61.873															267.18	1.20	70.00	1.485E-31	2617.934	7.248	74.886
ACTR2	1558015_s_at	ARP2 actin-related protein 2 homolog (yeast)	322.01	1.26	74.00	2.089E-43	3858.618	7.756	102.623															356.65	1.29	89.33	2.468E-49	17361.221	9.663	116.445
ACTR2	200727_s_at	ARP2 actin-related protein 2 homolog (yeast)	234.83	1.13	48.80	4.512E-19	170.214	4.362	45.648															275.53	1.15	57.33	3.831E-21	639.696	5.662	50.683
ACTR2	200729_s_at	ARP2 actin-related protein 2 homolog (yeast)															457.62	-1.12	50.64	3.764E-18	683.461	-5.548	44.086							
IFI16	208965_s_at	interferon, gamma-inducible protein 16	140.11	1.10	43.20	8.760E-18	107.415	3.868	41.937															161.40	1.11	46.67	5.054E-15	171.054	4.315	35.813
USP34	212065_s_at	ubiquitin specific protease 34	34.34	1.19	67.20	1.202E-40	2669.047	7.186	93.891															35.06	1.19	71.33	7.389E-27	3791.820	7.612	62.151
ROD1	207223_s_at	ROD1 regulator of differentiation 1 (S. pombe)	26.94	1.25	70.00	2.430E-39	2116.346	7.089	90.855															29.37	1.26	74.00	1.092E-27	3227.802	7.590	64.142
ROD1	214697_s_at	ROD1 regulator of differentiation 1 (S. pombe)	67.27	1.22	68.80	6.889E-45	3273.123	7.432	104.373															62.92	1.21	76.67	1.900E-34	5649.317	8.145	80.222
ROD1	214698_at	ROD1 regulator of differentiation 1 (S. pombe)	16.74	1.14	58.40	8.958E-40	2114.124	6.710	91.098															19.11	1.15	68.00	3.729E-32	5494.952	7.821	73.694
ROD1	224618_at	ROD1 regulator of differentiation 1 (S. pombe)	55.95	1.17	51.60	5.850E-17	122.823	4.169	39.644																					
FYB	205285_s_at	FYN binding protein (FYB-120/130)	314.21	1.28	64.40	4.132E-32	542.074	5.763	76.575															320.99	1.29	68.00	4.210E-23	803.330	6.203	55.895
FYB	211795_s_at	FYN binding protein (FYB-120/130)	434.38	1.17	64.00	2.266E-31	5260.901	7.682	74.866															437.27	1.18	67.33	1.119E-27	5812.650	7.870	66.370
TGFBR2	207334_s_at	transforming growth factor, beta receptor II (70/80kDa)	19.79	1.26	61.60	1.290E-29	523.734	5.661	68.224															20.92	1.27	66.00	2.149E-22	934.695	6.276	51.669
CD44	1557905_s_at	CD44 antigen (homing function and Indian blood group system)	358.91	1.16	49.20	1.918E-20	141.583	4.239	49.387															366.98	1.16	50.67	3.660E-14	173.255	4.433	34.957
CD44	204489_s_at	CD44 antigen (homing function and Indian blood group system)	408.54	1.18	53.60	1.032E-21	243.104	4.786	52.546															410.10	1.18	52.67	3.904E-14	196.390	4.585	35.089
CD44	210916_s_at	CD44 antigen (homing function and Indian blood group system)	210.38	1.20	53.60	2.711E-24	307.268	4.995	57.924															236.44	1.22	59.33	1.718E-19	645.997	5.764	47.070
CD44	212063_at	CD44 antigen (homing function and Indian blood group system)	461.63	1.08	37.20	1.995E-24	126.800	3.892	58.209															423.48	1.08	38.00	2.322E-15	114.467	3.819	37.160
CD44	216062_at	CD44 antigen (homing function and Indian blood group system)															0.77	1.11	51.28	4.083E-19	107.226	2.113	42.419							
LILRA1	207872_s_at	leukocyte immunoglob.like recep. subfam B (TM&ITIM domains) member 1	112.04	1.41	48.40	3.241E-23	122.644	4.267	55.311															107.91	1.39	48.67	1.641E-14	126.097	4.283	35.186
FOXO3	204131_s_at	forkhead box O3A (all values non-significant)	7.65	1.04	6.80	1.497E-01	7.623	1.428	2.004	14.19	-1.04	14.67	1.803E-01	17.103	-2.059	2.038	11.14	-1.02	2.56	5.702E-01	2.639	-0.666	0.775	3.35	1.02	3.33	5.184E-01	3.630	0.890	0.678
FOXO3	224889_at	forkhead box O3A (all values non-significant)	9.49	1.03	14.00	3.720E-02	12.470	1.819	3.523	8.34	-1.03	12.67	6.805E-02	11.033	-1.721	2.871	21.83	-1.07	29.49	6.013E-06	37.291	-2.818	12.891	8.56	1.02	16.67	1.236E-01	15.599	2.000	2.285
FOXO3	224891_at	forkhead box O3A (all values non-significant)	61.43	1.05	21.20	5.335E-04	22.678	2.334	8.955	40.81	-1.03	6.00	8.485E-02	5.787	-1.229	3.326	121.72	-1.11	34.62	5.304E-08	65.590	-3.351	19.330	49.30	1.03	18.67	2.951E-02	18.960	2.170	4.619
FOXO3	231548_at	forkhead box O3A (all values non-significant)	4.07	-1.06	14.00	4.683E-04	7.001	-0.801	7.912	0.47	1.01	0.67	5.600E-01	0.277	0.118	0.584	0.16	1.00	3.85	8.060E-01	1.493	0.167	0.216	4.25	-1.06	12.67	9.640E-03	5.860	-0.665	4.891
FOXO3	204132_s_at	forkhead box O3A	50.14	1.32	73.60	1.124E-44	2914.610	7.532	103.915															49.02	1.31	68.67	3.703E-23	1934.778	7.018	54.318
FOXO3	210655_s_at	forkhead box O3A	35.41	1.30	70.40	2.919E-39	850.408	6.319	91.015															36.66	1.33	72.00	7.494E-28	1047.305	6.159	64.836
STAT3	208992_s_at	signal transducer & activator of transcription 3 (acute-phase response fact.)	189.61	1.18	55.60	1.069E-22	289.850	4.968	54.032															196.55	1.18	56.00	5.194E-16	357.713	5.158	38.698
CYBB	203922_s_at	cytochrome b-245, beta polypeptide (chronic granulomatous disease)	416.70	1.16	54.80	1.993E-25	741.367	5.737	61.036															532.82	1.20	67.33	2.616E-26	3250.494	7.371	63.529
NLRP3	216015_s_at	cold autoinflammatory syndrome 1	52.85	1.29	68.40	1.702E-40	893.361	6.308	94.205															54.46	1.30	72.00	1.411E-29	1302.527	6.740	69.106
NLRP3	207075_at	cold autoinflammatory syndrome 1															100.47	-1.15	53.85	3.212E-18	1902.793	-6.507	42.942							
ATF7IP	218987_at	activating transcription factor 7 interacting protein	44.41	1.11	52.00	2.060E-21	156.361	4.334	49.374															54.74	1.14	54.67	5.163E-18	361.673	5.109	41.869
IL1RN	212659_s_at	interleukin 1 receptor antagonist	72.78	1.17	53.60	1.552E-21	201.337	4.621	50.431															67.23	1.16	51.33	3.799E-14	140.125	4.265	33.266
IL1RN	216243_s_at	interleukin 1 receptor antagonist	122.37	1.26	48.00	6.059E-21	107.380	4.060	49.846																					
ADAM10	202604_x_at	a disintegrin and metalloproteinase domain 10	49.32	1.17	51.60	1.820E-21	144.207	4.302	49.895															47.74	1.16	55.33	6.449E-15	218.060	4.710	34.719
ADAM10	214895_s_at	a disintegrin and metalloproteinase domain 10	80.25	1.29	53.60	8.664E-21	183.321	4.619	49.149															76.29	1.28	56.00	1.110E-13	201.920	4.740	32.690
CSF3R	1553297_a_at	colony stimulating factor 3 receptor (granulocyte)	354.85	1.26	51.20	4.982E-27	159.155	4.439	64.889															356.41	1.26	50.67	4.677E-17	154.127	4.400	41.906
NCF4	205147_x_at	neutrophil cytosolic factor 4, 40kDa	142.18	1.17	56.80	5.027E-24	340.747	5.124	56.775															137.70	1.17	56.00	1.575E-16	362.437	5.160	39.481
NCF4	207677_s_at	neutrophil cytosolic factor 4, 40kDa	124.51	1.14	46.40	3.616E-16	108.002	3.952	38.391															129.80	1.14	46.00	8.563E-13	124.934	4.067	30.677
RCC2	224578_at	regulator of chromosome condensation 2	129.23	-1.12	55.20	2.187E-40	2057.140	-6.584	94.034															124.49	-1.11	52.00	8.288E-23	1464.892	-6.210	53.453
BOLA2/2B	209836_x_at	bolA-like 2 (E. coli)	148.25	-1.25	75.20	5.452E-44	2049.917	-7.202	103.159																					
RRAGD	221523_s_at	Ras-related GTP binding D															52.65	1.20	41.03	4.135E-10	137.018	4.099	24.014							
RRAGD	221524_s_at	Ras-related GTP binding D															123.17	1.25	61.54	2.203E-19	1269.824	6.426	46.294							
NCR1	207860_at	natural cytotoxicity triggering receptor 1															3.58	1.25	66.03	4.928E-23	1518.857	5.098	51.966							
FCGR3A	204006_s_at	Fc fragment of IgG, low affinity IIIa//b, receptor (CD16a//b)															579.11	1.51	62.82	1.555E-10	617.592	5.990	28.218							
FCGR3B	204006_s_at	Fc fragment of IgG, low affinity IIIa//b, receptor (CD16a//b)															579.11	1.51	62.82	1.555E-10	617.592	5.990	28.218							
S1PR4	206437_at	endothelial differentiation, G-protein-coupled receptor 6															67.58	1.12	50.64	2.835E-14	396.459	5.090	33.330							
ADA	204639_at	adenosine deaminase															63.90	1.37	59.62	5.917E-16	1734.903	6.726	38.224							
ADA	216705_s_at	adenosine deaminase															41.33	1.40	69.87	7.477E-20	1008.913	4.232	46.851							
INSIG1	201625_s_at	insulin induced gene 1															30.83	1.29	64.74	7.679E-21	2483.967	7.124	48.512							
INSIG1	201627_s_at	insulin induced gene 1															75.10	1.21	57.05	8.520E-14	608.869	5.658	32.844							
CD83	204440_at	CD83 antigen (activated B lymphocytes, immunoglobulin superfamily)															28.29	1.27	53.21	9.887E-13	518.075	5.480	29.753							
CDC42EP4	218062_x_at	CDC42 effector protein (Rho GTPase binding) 4															15.40	1.12	48.08	3.336E-15	328.277	4.886	34.342							
ICAM4	207194_s_at	intercellular adhesion molecule 4, Landsteiner-Wiener blood group															34.51	1.86	66.67	6.635E-13	643.646	6.117	30.957							
TCF7L2	212759_s_at	transcription factor 7-like 2 (T-cell specific, HMG-box)															27.54	1.28	50.64	6.576E-13	223.317	4.432	30.056							
TCF7L2	212761_at	transcription factor 7-like 2 (T-cell specific, HMG-box)															313.96	1.31	44.87	3.593E-09	119.110	4.121	23.811							
TCF7L2	216035_x_at	transcription factor 7-like 2 (T-cell specific, HMG-box)															116.75	1.33	43.59	2.859E-09	111.815	4.064	23.132							
TCF7L2	216511_s_at	transcription factor 7-like 2 (T-cell specific, HMG-box)															82.66	1.36	49.36	3.188E-11	180.647	4.576	27.365							
IL21R	219971_at	interleukin 21 receptor															8.74	1.26	65.38	2.842E-18	166.202	2.034	41.554							
IL21R	221658_s_at	interleukin 21 receptor															13.98	1.41	54.49	1.068E-14	104.641	1.985	34.007							
IL21R	237753_at	interleukin 21 receptor															5.06	1.26	48.08	5.174E-10	183.521	4.222	22.219							
NOTCH4	205247_at	Notch homolog 4 (Drosophila)															12.63	1.16	51.28	4.283E-15	938.932	5.859	34.192							
PLAC8	219014_at	placenta-specific 8															564.45	1.24	41.67	1.733E-08	102.719	3.905	22.632							
IL2RG	204116_at	interleukin 2 receptor, gamma (severe combined immunodeficiency)															87.08	1.26	50.00	1.778E-12	251.614	4.802	30.110							
PDGFC	218718_at	platelet derived growth factor C															173.46	1.38	44.23	1.715E-08	116.380	4.136	21.902							
PDGFC	222719_s_at	platelet derived growth factor C															42.34	1.50	66.03	5.334E-22	2262.186	7.208	51.851							
GAS7	207704_s_at	growth arrest-specific 7															43.96	1.12	40.38	5.876E-12	138.680	4.041	27.622							
BRWD3	235156_at	Bromodomain and WD repeat domain containing 3															22.93	-1.22	73.08	1.051E-35	13796.183	-8.857	82.247							
WDFY3	212602_at	WD repeat and FYVE domain containing 3	26.87	1.13	46.00	4.438E-16	106.940	3.930	36.803															27.69	1.13	50.67	4.308E-13	147.139	4.275	29.961
WDFY3	212606_at	WD repeat and FYVE domain containing 3															75.07	-1.14	53.85	7.458E-17	882.335	-5.854	39.530							
USP32	206405_x_at	ubiquitin specific peptidase 32 /// ubiquitin specific peptidase 6															35.78	-1.25	51.92	2.337E-10	245.646	-4.809	24.294							
USP6	206405_x_at	ubiquitin specific peptidase 32 /// ubiquitin specific peptidase 6															35.78	-1.25	51.92	2.337E-10	245.646	-4.809	24.294							
BTAF1	209430_at	BTAF1 RNA polymerase II, B-TFIID transcription factor-associated, 170kDa															91.14	-1.21	58.97	2.792E-16	1178.975	-6.268	38.673							
DOCK5	219921_s_at	dedicator of cytokinesis 5								8.39	1.12	48.00	4.326E-16	196.918	4.459	36.036								14.10	1.22	63.33	1.662E-20	497.603	5.624	46.839
MAP2K7	226053_at	mitogen-activated protein kinase kinase 7	38.57	1.26	51.60	1.190E-17	112.167	4.151	41.134																					
MAP2K7	209951_s_at	mitogen-activated protein kinase kinase 7								7.57	1.13	50.67	1.867E-15	156.143	4.319	34.540														
JAK2	205842_s_at	Janus kinase 2 (a protein tyrosine kinase)								113.58	1.20	50.00	1.179E-10	115.911	4.114	25.927														
NGLY1	207492_at	N-glycanase 1								13.25	1.24	58.67	2.059E-17	482.658	5.374	39.785														
SNX14	230759_at	Sorting nexin 14								4.21	1.14	49.33	7.886E-11	106.183	3.879	23.696														
BTG3	205548_s_at	BTG family, member 3								11.11	1.21	52.00	2.900E-13	396.877	4.983	30.077														
SLAMF7	219159_s_at	SLAM family member 7								81.15	1.59	55.33	2.578E-09	340.784	5.357	23.866	97.03	1.70	63.46	6.067E-12	1000.522	6.534	30.185							
SLAMF7	222838_at	SLAM family member 7															126.27	1.55	48.08	6.047E-09	203.994	4.761	23.350							
IL10	207433_at	interleukin 10								6.77	1.18	44.67	7.825E-10	122.429	3.229	21.804														
NDUFAF2	228355_s_at	NADH dehydrogenase (ubiquinone) complex I, assembly factor 2								32.73	-1.13	43.33	4.843E-11	103.503	-3.866	25.370								43.49	-1.16	46.67	5.749E-13	116.175	-4.029	30.193
IL27RA	205926_at	interleukin 27 receptor, alpha								71.42	-1.16	64.00	4.467E-24	1928.484	-6.787	56.196														
IL27RA	222062_at	interleukin 27 receptor, alpha								105.78	-1.09	42.67	4.930E-11	101.152	-3.806	26.059														
IRF5	205468_s_at	interferon regulatory factor 5	73.86	1.37	48.00	5.629E-18	101.705	4.084	42.747															85.14	1.42	54.67	6.267E-15	207.671	4.830	35.979
TNFAIP3	202644_s_at	tumor necrosis factor, alpha-induced protein 3																						188.62	-1.19	54.00	4.601E-17	270.389	-4.891	41.184
TLR4	221060_s_at	toll-like receptor 4 /// toll-like receptor 4	130.83	1.20	54.00	5.602E-21	284.886	4.943	49.867															115.76	1.18	51.33	6.120E-14	178.339	4.484	33.430
TLR4	1552798_a_at	toll-like receptor 4																						93.79	1.16	54.00	2.852E-16	364.762	5.121	38.508
IL6R	217489_s_at	interleukin 6 receptor	92.61	1.44	55.60	5.265E-27	215.868	4.890	63.874															84.93	1.40	51.33	2.064E-16	137.769	4.411	39.307
IL6R	205945_at	interleukin 6 receptor /// interleukin 6 receptor								191.34	-1.11	42.00	1.684E-09	100.110	-3.800	23.199														
FOS	209189_at	v-fos FBJ murine osteosarcoma viral oncogene homolog	825.33	-1.48	48.00	1.002E-16	113.645	-4.238	42.794																					
PHF21A	1554153_a_at	PHD finger protein 21A	62.87	1.19	69.20	7.050E-48	4903.058	7.781	111.057															59.60	1.18	66.67	1.003E-30	4245.169	7.569	71.449
GLG1	207966_s_at	golgi apparatus protein 1	184.17	1.22	71.60	2.279E-38	4417.122	7.776	90.314															158.07	1.19	62.00	3.408E-21	1559.140	6.573	50.511
GLG1	214730_s_at	golgi apparatus protein 1	217.18	1.22	72.40	9.586E-48	2372.363	7.238	112.047															210.29	1.21	68.00	2.782E-31	2011.747	6.964	74.049
CCDC6	204716_at	coiled-coil domain containing 6	26.65	1.23	72.00	7.584E-55	4135.802	7.736	126.483															23.30	1.20	75.33	1.905E-36	5402.579	8.055	83.902
SF3A1	216457_s_at	splicing factor 3a, subunit 1, 120kDa	106.80	1.11	55.20	4.300E-50	2482.916	6.741	116.196								61.91	-1.06	30.13	4.653E-18	262.624	-4.320	41.458	98.54	1.10	54.00	3.712E-35	2323.320	6.646	81.652
TBCB	201804_x_at	cytoskeleton associated protein 1	123.92	-1.11	52.80	5.338E-33	2591.146	-6.709	76.898															125.71	-1.11	55.33	1.625E-21	2304.911	-6.677	50.461
TBCB	211759_x_at	cytoskeleton associated protein 1	94.23	-1.09	40.00	4.040E-16	153.267	-4.093	37.589															101.38	-1.09	46.67	1.754E-15	470.392	-5.132	36.235
TBCB	216194_s_at	cytoskeleton associated protein 1	76.64	-1.10	43.60	2.073E-22	156.283	-4.178	52.017															87.20	-1.11	47.33	4.370E-16	249.607	-4.634	37.639
GPR157	227970_at	G protein-coupled receptor 157	11.11	-1.13	56.80	5.401E-39	2447.981	-6.783	88.956															10.83	-1.13	52.00	7.892E-20	829.437	-5.745	44.796
RCC2	224578_at	regulator of chromosome condensation 2	129.23	-1.12	55.20	2.187E-40	2057.140	-6.584	94.034															124.49	-1.11	52.00	8.288E-23	1464.892	-6.210	53.453
BOLA2	210396_s_at	LAT1-3TM protein /// bolA-like 2 (E. coli) /// LAT1-3TM- like protein								122.00	-1.26	51.33	1.221E-11	184.468	-4.562	28.434								96.19	-1.21	44.67	3.380E-09	112.113	-4.003	22.362
ATG7	218673_s_at	APG7 autophagy 7-like (S. cerevisiae)	35.90	1.11	44.80	3.975E-23	117.367	3.975	53.137															38.95	1.12	44.67	1.773E-17	150.376	4.181	40.247
SORT1	212807_s_at	sortilin 1															70.17	1.17	65.38	3.871E-28	7973.885	8.084	65.606							
LSS	202245_at	lanosterol synthase (2,3-oxidosqualene-lanosterol cyclase)															43.96	1.18	64.74	2.850E-25	4155.963	7.498	58.632							
ACP5	204638_at	acid phosphatase 5, tartrate resistant															168.09	1.67	67.31	2.931E-20	3167.600	7.637	49.504							
DOCK8	232842_at	dedicator of cytokinesis 8															8.51	1.13	45.51	1.770E-10	120.651	4.022	23.137							
DOCK8	239027_at	dedicator of cytokinesis 8															70.70	1.24	46.15	4.060E-11	136.195	4.211	26.628							
CDKN1C	213182_x_at	cyclin-dependent kinase inhibitor 1C (p57, Kip2)															110.30	1.53	38.46	2.128E-08	100.645	4.003	21.587							
CDKN1C	213348_at	Cyclin-dependent kinase inhibitor 1C (p57, Kip2)															175.77	1.43	41.67	2.985E-07	100.311	4.001	19.286							
CDKN1C	216894_x_at	cyclin-dependent kinase inhibitor 1C (p57, Kip2)															112.31	1.45	42.31	7.006E-08	110.728	4.103	20.320							

Included are all Symbols, cited in figures or text of the paper. For comparison reasons 4 **FOXO3** IDs, never found significant were included to show the differences between significant and non-significant IDs. Not all values included in High-Performance Chip Data Analysis (HPCDA), are included in this table. Short explanations of the shown values: **Signal Differ**: Difference of averaged group one minus averaged group 2 of RMAExpress Quantile normalized Signals (Background corrected and summarized with Median Polish). **FC**: Fold Change between both groups. **% Incr - Decr**: Each Signal on chip of group 1 was compared with each Signal on chip of group 2 for all IDs; if a threshold was fulfilled this was counted as Increased (1 > 2) or Decreased (1 < 2) event, respectively; finally a percentage of maximal possible events results for both directions; here the absolute difference of these two percentages is shown. **ttest SLREvB vs. BvBEvE**: ttest of log2 of FCs (Signal Log Ratio, SLR) of group 1 (experiment group, EG or E) versus group 2 (baseline group, BG or B) , resulting in EvB, versus SLRs within both groups EG vs. EG (EvE) and BG versus BG (BvB); null hypothesis SLR EvB = 0 i.e. non-significant. **HPCDA-Score**: An empirical calculated score, optimized with Gene List Significance Index (GLSI), including all values calculated for HPCDA; explanations in DOI: 10.13140/RG.2.2.25762.38085 or DOI: 10.13140/RG.2.1.1426.8321. **HPCDA VolcanoX** and **Y**: An optimization of known Volcano Plot or Limma Volcano Plot with values of HPCDA; explanation in DOI: 10.13140/RG.2.1.2547.5923.
